# Blueberries and Honeysuckle Berries: Anthocyanin-Rich Polyphenols for Vascular Endothelial Health and Cardiovascular Disease Prevention

**DOI:** 10.3390/nu17243888

**Published:** 2025-12-12

**Authors:** Sanda Jurja, Ticuta Negreanu-Pirjol, Mihaela-Cezarina Mehedinți, Maria-Andrada Hincu, Bogdan-Stefan Negreanu-Pirjol, Florentina-Nicoleta Roncea, Alin Laurențiu Tatu

**Affiliations:** 1 Faculty of Medicine, “Ovidius” University of Constanta, 1, University Alley, Campus, Building B, 900470 Constanta, Romania; sanda.jurja@365.univ-ovidius.ro; 2 Faculty of Pharmacy, “Ovidius” University of Constanta, 6, Capitan Aviator Al. Serbanescu Street, Campus, Building C, 900470 Constanta, Romania; bogdan.negreanu@univ-ovidius.ro (B.-S.N.-P.); florentina.roncea@univ-ovidius.ro (F.-N.R.); 3 Academy of Romanian Scientists, Biological Sciences Section, 3, Ilfov Street, 050044 Bucharest, Romania; 4 Faculty of Medicine, “Dunarea de Jos” University of Galati, 800712 Galati, Romania; 5 Center of Research in Dermatology Science, Faculty of Medicine and Pharmacy, “Dunarea de Jos” University of Galati, 800712 Galati, Romania; alin.tatu@ugal.ro

**Keywords:** blueberries, haskap, *Lonicera caerulea*, *Vaccinium* spp., anthocyanins, cyanidin-3-O-glucoside, endothelial function, nitric oxide/eNOS, NRF2, NF-*κ*B23

## Abstract

Cardiovascular disease remains the world’s leading cause of death globally, and there is continuing interest in adjunct, diet-based strategies that may support vascular health alongside guideline-directed pharmacotherapy. Anthocyanin-rich berries are one such option: they are widely consumed, generally safe, and can provide substantial amounts of polyphenols in habitual diets. This narrative review focuses on two anthocyanin-rich species, blueberries (*Vaccinium* spp.) and haskap/blue honeysuckle (*Lonicera caerulea* L.), and examines the extent to which their intake may influence vascular endothelial function and cardiometabolic risk markers. For blueberries, which are typically dominated by malvidin- and delphinidin-based anthocyanins together with flavonols, phenolic acids and stilbenes such as pterostilbene, randomized controlled trials and meta-analyses have reported improvements in flow-mediated dilation, with modest effects on blood pressure and arterial stiffness in at-risk populations. Haskap berries, characterized by high levels of cyanidin-3-O-glucoside (C3G) and enriched in iridoids and vitamin C, have been studied mainly in cell and animal models, with early human data suggesting potential effects on vascular function, blood pressure and physical performance. Across both berries, emerging evidence indicates that vascular actions are mediated largely by gut- and host-derived phenolic metabolites rather than by transient circulating parent anthocyanins. We synthesize current knowledge on the phytochemical composition of blueberries and haskap, on molecular pathways implicated in endothelial protection (including NO/eNOS signaling, NRF2-mediated antioxidant defense, NF-κB-driven inflammation, lipoprotein metabolism and platelet activation), and on clinical outcomes related to vascular and cardiometabolic health. On this basis, we outline a mechanistic hypothesis that combined blueberry–haskap interventions could provide additive or synergistic effects on vascular function. This hypothesis is currently supported primarily by preclinical and indirect clinical evidence and should be regarded as hypothesis-generating, highlighting priorities for future mechanism-aware trials rather than constituting a practice-changing recommendation.

## 1. Introduction

Cardiovascular disease (CVD) remains the leading cause of morbidity and mortality worldwide and is strongly driven by modifiable lifestyle factors, including diet, physical inactivity, smoking, and metabolic disorders such as hypertension, dyslipidemia, and type 2 diabetes [1,2,3,4,5]. Endothelial dysfunction represents an early and at least partly reversible step in the atherosclerotic process and is increasingly recognized as a central therapeutic target for CVD prevention, as it integrates the impact of oxidative stress, inflammation, metabolic imbalance, and hemodynamic stress into measurable changes in vascular function [6,7,8,9,10]. Dietary strategies that can safely and sustainably support endothelial function therefore represent an important complement to pharmacological prevention and treatment, particularly when they provide bioactive compounds with pleiotropic vascular actions [10,11,12].

Within this context, anthocyanin-rich berries have attracted considerable interest because they combine high densities of (poly)phenols with generally favorable safety and palatability and can be incorporated into habitual dietary patterns [13,14,15,16]. Prospective cohort studies and randomized controlled trials suggest that higher intakes of anthocyanin-rich foods are associated with improved vascular function, lower blood pressure, and reduced risk of coronary events and CVD [12,17,18,19,20,21,22]. These effects appear to be mediated by multiple, interacting mechanisms, including enhanced nitric oxide (NO) bioavailability, attenuation of oxidative stress and inflammatory signaling, and modulation of platelet function, lipid handling, and cholesterol efflux [13,23,24,25,26,27,28,29,30,31,32]. At the same time, anthocyanin-rich foods differ markedly in their phytochemical profiles, bioavailability, and matrix characteristics, and these differences may translate into distinct vascular effects [14,16,21,33,34,35,36,37,38,39,40]. In parallel, processing and formulation technologies—including drying methods, co-pigmentation, encapsulation, and polymer-based delivery systems—are being developed to enhance anthocyanin stability and bioavailability in berry-derived products [34,35,40,41,42,43,44,45].

Blueberries (*Vaccinium* spp.) are among the most widely consumed anthocyanin-rich berries and have been extensively investigated in both preclinical models and human intervention trials for their potential cardiometabolic and vascular benefits [13,15,16,21,33,34,46,47,48,49]. Blueberries typically contain a complex mixture of anthocyanins dominated by malvidin and delphinidin glycosides, together with other flavonoids, phenolic acids, and stilbenes such as resveratrol and pterostilbene [13,16,50,51,52,53,54]. Clinical trials in various populations, ranging from healthy individuals to those with metabolic syndrome or elevated cardiometabolic risk, have reported improvements in surrogate markers of vascular health—including flow-mediated dilation (FMD), blood pressure, arterial stiffness, and circulating biomarkers of endothelial activation—after blueberry intake [17,21,22,55,56,57,58,59,60,61,62,63]. Nevertheless, not all studies have shown consistent benefits, and heterogeneity in dose, formulation, study duration, and population characteristics complicates the translation of these findings into clear, evidence-based dietary recommendations [12,17,20,46,47,49].

Haskap or blue honeysuckle berries (*Lonicera caerulea* L.) represent a comparatively less explored but promising anthocyanin-rich berry species with increasing horticultural and nutritional relevance [64,65,66,67,68,69]. Haskap is characterized by very high levels of cyanidin-3-O-glucoside (C3G) as a predominant anthocyanin, alongside other anthocyanins, flavonols, phenolic acids, and a distinctive profile of iridoids and related secoiridoid glycosides [66,70,71,72,73,74,75,76]. These compositional features confer potent antioxidant, anti-inflammatory, and vasomodulatory properties *in vitro* and *in vivo*, with emerging data on improvements in oxidative stress, blood pressure, and vascular function in experimental models and small human studies [64,69,77,78,79,80,81]. However, the clinical evidence base for haskap remains limited to a small number of acute and short-term interventions, and most mechanistic insights are still derived from cell and animal models, with only early trials and ongoing studies beginning to address vascular endpoints in older adults and at-risk populations [69,80,82,83,84,85].

The different but overlapping phytochemical profiles of blueberries and haskap raise the hypothesis that their combination might offer complementary or even synergistic effects on vascular endothelial health by targeting converging and distinct pathways [13,14,33,52,53,64,66,69,70,71,73,74,84]. Blueberries contribute a diverse anthocyanin spectrum and additional bioactives such as pterostilbene, whereas haskap provides high C3G content and unique iridoids with anti-inflammatory and vasomodulatory potential, suggesting a multi-layered model of mechanistic complementarity rather than simple redundancy [13,14,52,53,66,69,70,71,72,73,74,75,76,84]. The broader concept of synergy between natural-product polyphenols is supported by work on multi-component interventions and formal models of combination effects, indicating that appropriately designed mixtures may achieve greater vascular protection than individual compounds alone [86,87,88]. At the same time, anthocyanins and related (poly)phenols exhibit low systemic bioavailability, and their vascular effects are thought to be mediated to a large extent by phenolic metabolites generated through gut microbial metabolism [21,27,29,30,36,37,38,89,90,91]. Inter-individual differences in gut microbiota composition and function may therefore modulate the vascular response to berry intake, giving rise to distinct “(poly)phenol metabotypes” and contributing to variability in clinical outcomes [21,27,36,92,93].

The present review aims to provide an integrated, mechanism-oriented overview of how blueberries and haskap berries may support vascular endothelial health and contribute to CVD prevention. Specifically, we (i) summarize and compare the phytochemical composition of blueberries and haskap with a focus on anthocyanins and other bioactives relevant to vascular function; (ii) review preclinical evidence on molecular pathways involved in endothelial protection, oxidative stress, inflammation, platelet function, and lipid handling; and (iii) critically evaluate the available human data on vascular and cardiometabolic outcomes. On this basis, we discuss a hypothesis of mechanistic complementarity and potential synergy between blueberries and haskap while explicitly acknowledging the imbalance between the relatively robust clinical evidence for blueberries and the predominantly preclinical evidence for haskap, and we highlight key gaps—including bioavailability, inter-individual variability, safety, and long-term efficacy—that should be addressed in future mechanism-aware clinical trials. Compared with previous reviews on berries, blueberries or haskap, this article integrates both species into a single vascular-focused framework, updates the evidence base with recent randomized trials and mechanistic studies (through October 2025), and explicitly links phytochemical profiles, metabolites and clinical endpoints. In addition, we move beyond descriptive summaries by outlining concrete, mechanism-aware clinical trial designs to test the hypothesized additive and synergistic effects of combined blueberry–haskap interventions.

## 2. Materials and Methods

### 2.1. Literature Search Strategy

This narrative review is based on a structured literature search focusing on blueberries (*Vaccinium* spp.), haskap/blue honeysuckle (*Lonicera caerulea* L.), anthocyanin-rich polyphenols, and vascular or cardiovascular outcomes. Electronic searches were conducted in PubMed/MEDLINE, Scopus, and Web of Science from database inception to the last search update on 4 October 2025.

The core search strategy combined three concept blocks: (i) plant sources (blueberries and haskap), (ii) anthocyanins and other berry (poly)phenols, and (iii) vascular or cardiometabolic endpoints. Examples of keyword combinations included: “blueberry” OR “blueberries” OR “*Vaccinium*” AND “haskap” OR “blue honeysuckle” OR “*Lonicera caerulea*” AND “anthocyanin*” OR “polyphenol*” AND “endothelial function” OR “vascular function” OR “flow-mediated dilation” OR “arterial stiffness” OR “blood pressure” OR “cardiovascular” OR “cardiometabolic”.

Additional searches were performed using terms related to mechanistic endpoints (e.g., “nitric oxide”, “eNOS”, “NF-κB”, “oxidative stress”, “platelet aggregation”) and to bioavailability and metabolism (e.g., “anthocyanin metabolites”, “phenolic acids”, “gut microbiota”). Reference lists of key publications and relevant reviews were manually screened to identify additional primary studies (snowballing). No lower date limit was imposed, and studies were eligible regardless of publication year as long as they met the inclusion criteria described below.

### 2.2. Eligibility Criteria

We included peer-reviewed articles in English that met at least one of the following criteria:

(1) Phytochemical composition studies characterizing the bioactive profile of blueberries (*Vaccinium* spp.) or haskap/blue honeysuckle (*Lonicera caerulea* L.), with an emphasis on anthocyanins, other (poly)phenols, and secondary metabolites relevant to vascular health.
(2) *In vitro* or *in vivo* preclinical studies investigating mechanistic effects of blueberries, haskap, or their isolated (poly)phenolic constituents on endothelial function, vascular signaling pathways, oxidative stress, inflammation, platelet function, lipid handling, or related cardiometabolic endpoints.
(3) Human observational studies or intervention trials (acute or chronic) assessing vascular, hemodynamic, or cardiometabolic outcomes after intake of blueberries, haskap, or their extracts, including outcomes such as flow-mediated dilation (FMD), arterial stiffness, pulse wave velocity, blood pressure, biomarkers of endothelial activation, or composite cardiovascular risk markers.

Narrative reviews and meta-analyses were used to contextualize the evidence and to identify additional primary studies but were not the primary units of data extraction. We excluded conference abstracts, non-peer-reviewed reports, case studies, animal experiments unrelated to vascular or cardiometabolic endpoints, and articles not providing sufficient methodological detail or outcome data. Studies not available in English were not considered.

### 2.3. Study Selection and Data Extraction

Titles and abstracts identified through the database searches were screened for relevance to blueberries, haskap, anthocyanins, and vascular or cardiometabolic outcomes. Potentially eligible articles were retrieved in full text and assessed against the inclusion and exclusion criteria described above. When multiple reports from the same study population were identified, we prioritized the most complete or recent publication.

For each included article, we extracted qualitative information on: plant material and preparation (whole berries, juices, extracts, purified compounds), dose and duration (for *in vivo* and clinical studies), study design and population (for human trials), primary vascular or cardiometabolic endpoints, and key mechanistic findings (e.g., effects on nitric oxide bioavailability, oxidative stress, inflammatory signaling, or platelet function). For phytochemical studies, we recorded the main classes and representative compounds (e.g., predominant anthocyanins, flavonols, phenolic acids, iridoids, stilbenes) and their reported concentration ranges.

### 2.4. Approach to Evidence Synthesis

Given the broad mechanistic and translational scope of the topic, we adopted a narrative synthesis rather than a quantitative meta-analysis. Evidence was organized into three main domains: (i) phytochemical composition of blueberries and haskap, (ii) preclinical mechanisms relevant to vascular endothelial function and cardiovascular risk, and (iii) clinical and cardiometabolic outcomes in human populations. Within each domain, we qualitatively compared findings across studies, with particular attention to dose, duration, population characteristics, and methodological differences that might explain heterogeneity in outcomes.

Because the included studies spanned diverse designs (from cell-based experiments to randomized controlled trials), formal risk-of-bias scoring using a single tool was not feasible. Instead, we comment qualitatively on key methodological strengths and limitations where relevant (e.g., sample size, control conditions, blinding, biomarker selection). The proposed hypothesis of synergistic vascular benefits from combining blueberries and haskap is therefore grounded in a qualitative integration of phytochemical profiles, mechanistic evidence, and the existing—albeit imbalanced—clinical literature, and should be interpreted accordingly as a hypothesis-generating framework.

## 3. Bioactive Principles Composition of Honeysuckle Berries/Haskap (*Lonicera caerulea* L.)

### 3.1. General Phytochemical Profile

Blueberries (*Vaccinium* spp.), members of the Ericaceae family, are globally recognized as one of the richest natural sources of bioactive phytochemicals. Their phytochemical complexity includes diverse classes of compounds such as flavonoids (anthocyanins, flavonols, flavan-3-ols), phenolic acids, tannins, and stilbenoids, alongside vitamins (notably C and K), minerals (particularly manganese), and dietary fiber [13,14,43,50]. This rich composition underlies their classification as a functional food with wide-ranging effects on human health, especially cardiovascular protection [13,43,50].

Compositional analyses consistently reveal that blueberries contain higher concentrations of anthocyanins compared to many other fruits, often cited as nature’s most potent antioxidant pigments [4,14,15]. These anthocyanins contribute significantly to the fruit’s total antioxidant capacity (TAC), which positions blueberries among the top-ranked fruits in terms of radical scavenging activity and oxidative stress modulation [24,51,56]. In addition, flavonols such as quercetin and myricetin, catechins, and hydroxycinnamic acids contribute synergistically to this antioxidant profile [25,26]. The phytochemical profile of blueberries is not limited to antioxidants alone. Beyond radical scavenging, blueberry polyphenols influence endothelial signaling, glucose homeostasis, lipid metabolism, and neuroprotection [17,47,94,95]. Mechanistic and clinical studies show that blueberry polyphenols enhance endothelial function and attenuate vascular inflammatory signaling [48,57,58]. Randomized trials further report improved insulin sensitivity and favorable changes in cardiometabolic profiles following blueberry intake in adults with metabolic risk [11,58]. Recent reviews have emphasized that the health-promoting activity of blueberries cannot be attributed to anthocyanins alone but rather to the interplay among multiple polyphenolic classes, micronutrients, and their metabolites [59]. Consistently, blueberry interventions improve vascular function in both preclinical models and human trials, and several studies also report reductions in oxidative stress—underscoring the translational relevance of their bioactive composition [9,49,59].

### 3.2. Anthocyanins as Key Bioactive Compounds

Anthocyanins are the predominant flavonoid subclass in blueberries and are primarily responsible for their deep blue–purple coloration. More than 20 distinct anthocyanin glycosides have been identified in blueberry species, with malvidin-, delphinidin-, cyanidin-, petunidin-, and peonidin-derived forms being the most abundant [13,14,16,23,43,50]. Among these, malvidin-3-glucoside and malvidin-3-galactoside are consistently reported as dominant across multiple cultivars, often contributing the largest share of total anthocyanin content [5,14,16,23]. The relative distribution of anthocyanins is influenced by species and ripening stage, with marked variability across cultivated highbush (*Vaccinium corymbosum*), wild lowbush (*Vaccinium angustifolium*), and bilberry (*Vaccinium myrtillus*) [14,16,23,50]. Wild lowbush and bilberry generally exhibit higher total anthocyanin concentrations than cultivated highbush, a feature that likely contributes to the robust biological activity reported for their extracts in experimental and human studies [14,23,49,50]. Beyond their pigmenting role, anthocyanins exert a broad spectrum of biological effects relevant to vascular health. Mechanistically, they act as antioxidants and signaling molecules, lowering reactive oxygen species and supporting endogenous antioxidant defenses while engaging endothelial pathways [25,26]. Malvidin glycosides and related anthocyanins increase nitric oxide (NO) bioavailability via PI3K/Akt–eNOS signaling and may modulate xanthine oxidase activity [26,94]. These mechanisms are consistent with improvements in endothelium-dependent vasodilation and modest reductions in diastolic blood pressure reported with blueberry interventions [17,25,26]. Anthocyanins also exhibit strong anti-inflammatory properties by modulating redox-sensitive transcription factors. Both *in vitro* and *in vivo* evidence shows that malvidin-3-glucoside and malvidin-3-galactoside suppress NF-κB activation, leading to the lower expression of pro-inflammatory mediators (e.g., TNF-α, ICAM-1, VCAM-1, MCP-1) [25,46]. This dual antioxidant and anti-inflammatory action provides a mechanistic basis for the vascular benefits associated with blueberry intake. In humans, blueberries improve endothelium-dependent vasodilation in both acute and longer-term settings [24,58,60]. In addition, anthocyanin-rich interventions—including mixed-berry diets and purified anthocyanins—have been shown to attenuate platelet activation/aggregation [61,96,97]. Finally, reductions in circulating oxidized LDL have been reported in adults with metabolic syndrome following blueberry supplementation, although findings are not universal across protocols [98]. These processes—enhanced endothelial function, tempered platelet reactivity, and improved lipoprotein redox status—are all central to atherogenesis and align with broader polyphenol mechanisms [11,57,58,61,96,97,98]. Recent clinical evidence supports these mechanistic findings. A 2024 systematic review and meta-analysis of randomized trials reported that blueberry interventions increased FMD by 1.50 percentage points (95% CI 0.81–2.20) and reduced diastolic blood pressure by about 2.2 mmHg (MD −2.20 mmHg; 95% CI −4.13 to −0.27), with larger blood-pressure reductions observed in smokers (SBP −3.92 mmHg; DBP −2.20 mmHg) [17]. Moreover, subgroup analyses suggest a dose-dependent pattern by anthocyanin intake, with greater FMD gains at higher anthocyanin doses, supporting a contributory role for anthocyanins and their metabolites in blueberry-associated vascular benefits [17]. In summary, blueberry anthocyanins constitute a structurally diverse group of bioactives with multifunctional properties. Their antioxidant, anti-inflammatory and vasodilatory actions, combined with their high abundance in the fruit, position them as prominent contributors to the cardioprotective profile of blueberries. Future studies should compare anthocyanin profiles across cultivars, optimize processing to preserve these compounds, and delineate the relative contributions of individual anthocyanins and their metabolites to vascular outcomes [17].

### 3.3. Other Polyphenols and Secondary Phytochemicals

Although anthocyanins dominate the phytochemical profile of blueberries, other polyphenols and secondary metabolites substantially contribute to their overall bioactivity. Flavonols such as quercetin, myricetin, and kaempferol are consistently detected in blueberry extracts, typically in glycosylated forms [13,43,50]. These flavonols exhibit potent antioxidant activity and can modulate endothelial nitric oxide synthase (eNOS) and nitric oxide (NO) bioavailability, thereby supporting vasodilation [14,46,55]. Quercetin, in particular, has been widely studied for its ability to inhibit platelet aggregation and downregulate inflammatory cytokines, complementing anthocyanins in vascular protection [46,55]. Flavan-3-ols, including catechin and epicatechin, represent another subclass of blueberry polyphenols, albeit at lower abundance than anthocyanins [16,23]. These compounds—also present in tea and cocoa—exhibit antioxidant and anti-inflammatory activities and have been implicated in vascular redox signaling, including attenuation of NADPH oxidase-derived ROS and engagement of NRF2-dependent defenses [46,55]. Within the blueberry matrix, flavan-3-ols likely act in concert with anthocyanins to support endothelial homeostasis and mitigate oxidative stress, a pattern consistent with improvements observed in human trials with whole-blueberry interventions [24,46,55]. Phenolic acids, especially chlorogenic, caffeic, ferulic, and protocatechuic acids, are present in blueberries and contribute to the overall (poly)phenolic profile [13,14,16,23,50]. Chlorogenic acid (caffeoylquinic acid) is frequently reported at relatively high levels in berry matrices and has been linked—primarily in preclinical and integrative reviews—to improvements in glucose handling and favorable modulation of lipid metabolism [94]. Phenolic acid metabolites derived from anthocyanin catabolism, notably protocatechuic acid, exert anti-inflammatory and anti-atherogenic actions, extending the biological impact of blueberry consumption [47,94]. Consistent with these mechanisms at the whole-food level, clinical syntheses report improvements in endothelial function following blueberry interventions [17]. Proanthocyanidins (condensed tannins)—oligomeric and polymeric flavan-3-ols—are also detected in blueberries, albeit at lower abundance than anthocyanins [14,23,50,95]. These compounds exhibit notable radical-scavenging capacity, can inhibit LDL oxidation in experimental systems, and are increasingly linked to shifts in gut microbiota composition with potential vascular relevance [57,95]. Finally, pterostilbene—a stilbenoid structurally related to resveratrol—is also reported in blueberries [14]. Compared with resveratrol, pterostilbene is often described as more lipophilic and potentially more orally bioavailable, a property sometimes invoked to explain its biological activity (overview in [59]). Preclinical and early clinical literature summarized in [48] associates pterostilbene with lipid-lowering, anti-inflammatory, and antidiabetic actions, though evidence remains heterogeneous and context-dependent. Within the whole-food matrix, human trials with blueberries demonstrate vascular benefits such as improved endothelium-dependent vasodilation, consistent with a contributory role of multiple constituents beyond anthocyanins [58,59]. Taken together, these non-anthocyanin polyphenols and secondary metabolites enhance the overall bioactivity of blueberries. Their complementary roles in antioxidant defense, vascular modulation, metabolic regulation, and anti-inflammatory activity suggest that cardioprotective effects arise from the synergistic interplay of multiple phytochemicals [46,55,57,59,94,95].

### 3.4. Factors Influencing Bioactive Content

The concentration and composition of bioactive compounds in blueberries are highly variable and shaped by genetic, environmental, and post-harvest factors. Species and cultivar are primary determinants of anthocyanin and broader polyphenol levels. For example, wild lowbush (*Vaccinium angustifolium*) and bilberry (*Vaccinium myrtillus*) frequently display higher total anthocyanin concentrations than cultivated highbush (*Vaccinium corymbosum*) or rabbiteye (*Vaccinium virgatum*) [14,50]. These differences are not merely quantitative: anthocyanin profiles also differ, with bilberry showing a distinct distribution of glycosylated anthocyanins relative to highbush cultivars [14,50]. It has been proposed that such interspecific variation may contribute to the more robust biological effects sometimes reported for wild species compared with their cultivated counterparts [55]. Environmental conditions during growth also play a crucial role. Factors such as sunlight/UV exposure, temperature regime, altitude, and soil characteristics modulate anthocyanin biosynthesis in *Vaccinium* spp. Cooler climates and greater UV exposure are frequently associated with higher anthocyanin accumulation, whereas excessive heat can depress flavonoid synthesis [14,50]. Developmental (ripening) stage further shapes these patterns [16]. Consistently, seasonal and geographical variability in anthocyanin content has been documented across blueberry-producing regions, reflecting genotype × environment interactions [14,50]. Maturity stage at harvest is another determinant of phytochemical content. Anthocyanin concentrations rise markedly during ripening, peaking at full blue coloration [16]. In contrast, some non-anthocyanin phenolics (e.g., selected hydroxycinnamic acids and flavonols) can decline slightly at advanced maturity, consistent with shifts in phenylpropanoid allocation [14,16,23,50]. These patterns indicate that both harvest timing and intended use (fresh consumption vs. processing) should be tailored to maximize target bioactive yields [14,23]. Post-harvest handling and processing methods significantly affect the retention of bioactive compounds. Freezing is generally effective at preserving anthocyanins and phenolic acids over extended storage and is the most widely used approach to extend blueberry shelf life [23]. Lyophilization (freeze-drying) is often considered the benchmark for preserving the overall phytochemical profile, maintaining higher antioxidant capacity than conventional convective drying [34]. In contrast, thermal treatments (e.g., pasteurization, hot-air drying) can cause substantial anthocyanin degradation in a time–temperature-dependent manner [33,34,35]. Even under optimized storage, gradual losses of anthocyanins and vitamin C are observed, underscoring that both temperature and duration are critical determinants of retention [23]. Processing for juice, purées, or powders introduces additional complexity. While juices and concentrates often retain a portion of phenolic acids, they typically lose a substantial fraction of anthocyanins due to thermal exposure, oxygen ingress, and enzymatic/oxidative degradation [23,33,34]. Powders derived from freeze-dried blueberries generally preserve bioactive compounds more effectively; however, particle size, co-/microencapsulation, co-pigmentation, and storage conditions (temperature, humidity, oxygen, time) further modulate stability [33,34,35,41]. These technological aspects are increasingly relevant as consumer demand for blueberry-based nutraceuticals and functional foods grows [33,35]. In conclusion, the phytochemical richness of blueberries is shaped by a multifactorial interplay of species genetics, environmental influences, harvest maturity, and processing conditions. Understanding and optimizing these factors are essential not only to maximize the nutritional value of fresh fruit but also to ensure that processed blueberry products retain their health-promoting potential [13,16,23,33,34,35,50].

### 3.5. Bioavailability and Circulating Metabolites

Despite their high concentrations in blueberries, native anthocyanins exhibit relatively low systemic bioavailability. Human feeding studies consistently show that <1% of ingested anthocyanins appear in plasma or urine in their intact glycosylated form [89,92,93,99]. This apparent paradox—high dietary intake but low direct absorption—has fueled interest in the metabolites generated during digestion, microbial fermentation, and host metabolism. Following ingestion, anthocyanins undergo extensive transformation. In the stomach and small intestine, limited absorption of intact anthocyanins occurs, while deglycosylation, followed by phase-II conjugation (glucuronidation, sulfation, methylation) proceeds in enterocytes and the liver [14,89,92]. A substantial fraction reaches the colon, where the gut microbiota cleaves the heterocyclic ring to yield lower-molecular-weight phenolic acids (for example, protocatechuic, vanillic, syringic, ferulic acids) [89,92,93]. These metabolites display greater stability and bioavailability than their parent compounds and persist in circulation for hours after consumption [89,92,93]. Converging evidence indicates that these phenolic metabolites, rather than intact anthocyanins, are key mediators of vascular and metabolic benefits associated with blueberry intake [55,92,93]. Consistent with this, both cell and clinical data show modulation of inflammatory and endothelial pathways relevant to vascular function [24,51,56]. In controlled human studies, plasma concentrations of anthocyanin-derived metabolites rise within 1–2 h of blueberry intake and can remain detectable up to 24 h, depending on dose and matrix [89,92]. Longer-term randomized trials also report improvements in endothelial function with sustained blueberry consumption [24]. The gut microbiome plays a pivotal role in shaping the profile and abundance of anthocyanin-derived metabolites. Inter-individual differences in microbial composition are linked to distinct metabolite patterns and may underlie variability in physiological responses to blueberry intake [92,95]. Overall, anthocyanins should be viewed not only as direct antioxidants but as precursors within a metabolic network that generates more bioavailable phenolic acids with capacity to modulate oxidative stress, inflammation, and vascular tone [55,89,92,93].

## 4. Bioactive Principles Composition of Honeysuckle Berries/Haskap (*Lonicera caerulea* L.)

### 4.1. General Phytochemical Profile

Honeysuckle berries (*Lonicera caerulea* L.), also known as haskap or honeyberry, are exceptionally rich in bioactive phytochemicals with nutritional and pharmacological relevance. Their phytochemical profile comprises anthocyanins, flavonols, flavan-3-ols, phenolic acids, iridoids, vitamin C, vitamin E, minerals, and dietary fiber [62,64,65,71]. The antioxidant potential of these fruits is reported to be three- to fivefold higher than that of more commonly consumed berries, justifying their classification as a “superfruit” [72]. The phytochemical composition of *Lonicera caerulea* L. (haskap) exhibits remarkable variability depending on genotype, cultivar, and environmental conditions. Comparative analyses of ten Polish cultivars revealed significant differences in both the qualitative and quantitative profiles of phenolic compounds, with anthocyanins representing up to 92% of total polyphenols in some genotypes [100]. Furthermore, the concentration and distribution of phenolics and iridoids are strongly affected by fruit morphology: the skin is particularly rich in anthocyanins, kaempferol and iridoid derivatives, while the pulp predominantly contains quercetin glycosides, sugars, and organic acids [73]. Similar compositional trends have been confirmed across European and Asian cultivars, with substantial regional variability in total phenolic content, anthocyanin concentration, and antioxidant capacity [64,74,78]. These variations are attributed to genetic diversity, climatic conditions, soil composition, and cultivation practices [62,71]. The consistent presence of bioactive phenolics—especially anthocyanins, flavonols, and iridoids—underpins the growing interest in *L. caerulea* as a source of functional ingredients for nutraceutical and therapeutic formulations [72].

### 4.2. Anthocyanins as Key Bioactive Compounds

In *Lonicera caerulea* (haskap), anthocyanins constitute the predominant fraction of polyphenols. The principal anthocyanin is cyanidin-3-O-glucoside (C3G), typically comprising 79–93% of the total anthocyanin pool, with other constituents including cynidin-3-rutinoside, cyanidin-3,5-diglucoside, pelargonidin-3-glucoside, and peonidin glycosides [65,66,67]. Across cultivars, maturity stages, and environments, total anthocyanin concentrations commonly range from 150 to >650 mg/100 g fresh weight (f.w.) [66,67,75,78]. C3G shows limited stability, being highly sensitive to pH and temperature; thermal processing accelerates its breakdown into protocatechuic acid (PCA) and phloroglucinaldehyde (PGA), metabolites with biological activities of their own [101]. Functionally, haskap anthocyanins—particularly C3G—exert antioxidant and anti-inflammatory actions and improve metabolic endpoints by engaging AMPK and PPAR-α signaling [102,103]. *In vivo*, C3G rich/haskap extracts attenuate hepatic steatosis, oxidative stress, and inflammatory markers, while haskap also inhibits pancreatic lipase activity and modulates the gut microbiota in diet-induced obesity models [102].

### 4.3. Other Polyphenols and Secondary Phytochemicals

Beyond anthocyanins, *Lonicera caerulea* (haskap) provides a broad spectrum of polyphenols and secondary metabolites. HPLC profiling consistently detects flavonols—especially quercetin, rutin, and kaempferol derivatives—which contribute to antioxidant and anti-inflammatory activity [64,79,104]. Flavan-3-ols (catechin, epicatechin) and proanthocyanidins are also present; compositional studies identify proanthocyanidins among the predominant constituents alongside catechins and rutin [64,75,79]. Preclinical work with *L. caerulea* extracts shows vascular protection consistent with reduced aortic NADPH-oxidase expression and increased vascular NOS activity, and polyphenol-dependent anti-inflammatory effects have likewise been demonstrated [104,105]. A distinctive feature of haskap is its abundance of iridoids—notably loganic acid (predominant), loganin, sweroside, and seco-loganin—with totals typically around 120–300 mg/100 g fresh weight and cultivar-dependent maxima reported near 276–372 mg/100 g f.w. [71,72]. Among phenolic acids, chlorogenic acid (5-O-caffeoylquinic acid) is the leading hydroxycinnamate; studies on Romanian cultivars corroborate this pattern and link these extracts to anti-lipid-droplet activity in hepatocyte models [74,75]. Vitamin C varies widely across cultivars and environments (17–186 mg/100 g f.w.) and, together with vitamin E and flavonols (e.g., rutin), contributes to total antioxidant capacity, with reports of synergistic interactions within the berry matrix [64,72].

### 4.4. Factors Influencing Bioactive Compounds Content

The phytochemical richness of *Lonicera caerulea* (haskap) is strongly shaped by cultivar and genotype. Recently profiled cultivars such as ‘Wulan’ and ‘Lanjingling’ display very high cyanidin-3-O-glucoside (C3G) levels (e.g., 471 mg/100 g f.w. for ‘Wulan’), under-scoring pronounced genotypic effects [65]. Multi-cultivar surveys likewise report marked genotype- and origin-dependence of phenolic markers (e.g., C3G, rutin, chlorogenic acids) [64,66]. Environmental conditions further modulate these profiles. Cross-site comparisons indicate that temperature and solar radiation are key drivers; higher sunshine is associated with higher vitamin C, while location and harvest stage affect anthocyanins and total phenolics [62,64]. Genome-scale analyses provide a mechanistic basis for high C3G content in *L. caerulea*. Notably, three anthocyanin 3,5-O-methyltransferases (LcOMT2/14/20) and two 3-O-glycosyltransferases (including LcUGT78x1 implicated in C3G accumulation) have been identified as central to haskap anthocyanin biosynthesis [80]. Agronomic practices and “terroir” (site/soil/cultivation regime) also contribute to between-orchard variability in phenolic and iridoid profiles, as shown by comparative work across locations and targeted chemical profiling of regional cultivars [64,66,74]. Post-harvest and processing steps determine the stability of bioactives. Non-thermal preservation (e.g., freezing, freeze-drying) is generally more protective of the antioxidant profile than conventional heating, whereas thermal treatments accelerate anthocyanin degradation—with the formation of protocatechuic acid (PCA) and phloroglucinaldehyde (PGA)—altering color and potential bioactivity [64,67].

### 4.5. Bioavailability and Circulating Metabolites

Despite their high concentrations in the fruit, native anthocyanins from *Lonicera caerulea* (haskap) show limited systemic bioavailability: intact glycosides typically account for <1% of dose recovered in plasma or urine, whereas the most absorbed label appears as downstream phenolic metabolites [76,99,106]. During digestion and microbial transformation, cyanidin-3-O-glucoside (C3G) yields protocatechuic acid (PCA) and related benzoic-acid derivatives (e.g., vanillic acid), and—depending on the matrix and co-occurring phenolics—caffeic/ferulic-type acids, which are more stable and can act as bioactive surrogates of the parent anthocyanin [36,76,106]. These circulating metabolites modulate oxidative-stress and inflammatory signaling, including AMPK activation and NF-κB/Nrf2 pathways, consistent with mechanistic data from haskap extracts and anthocyanin-metabolite studies [76,101,107]. Inter-individual variability in responses is strongly influenced by the gut microbiota, which shapes metabolite profiles and physiological effects; notably, haskap extracts also modulate the microbiome *in vivo* [76,102]. Finally, in haskap, the co-occurrence of anthocyanins with iridoids (e.g., loganic acid) likely provides complementary antioxidant/anti-inflammatory actions compared with berries lacking iridoids, supporting a network view of bio-efficacy rather than reliance on native anthocyanins alone [37,64,71]. Taken together, the health effects of haskap arise from both native anthocyanins and their phenolic derivatives, which offer a mechanistic basis for proposed nutraceutical and early clinical applications [64,76].

### 4.6. Comparative Phytochemistry

Blueberries (*Vaccinium* spp.) and haskap (*Lonicera caerulea* L.) share a high density of anthocyanins and other polyphenols, but differ in their dominant anthocyanin patterns. In blueberries, malvidin- and delphinidin-based glycosides are typically the most abundant, with petunidin, cyanidin and peonidin derivatives also present [16,33,50,90]. Blueberries also contain substantial flavonols (quercetin, myricetin, kaempferol), flavan-3-ols (catechin, epicatechin), phenolic acids (notably chlorogenic and caffeic acids), proanthocyanidins, and the stilbenoid pterostilbene [23,50,52]. In contrast, haskap shows a cyanidin-centric profile in which cyanidin-3-O-glucoside (C3G) often dominates the anthocyanin pool (frequently 80–90% of total), with cyanidin-3-rutinoside and cyanidin-3, 5-diglucoside as notable co-constituents; delphinidin/peonidin derivatives are generally minor contributors relative to C3G [62,65,66,70]. Beyond anthocyanins, haskap provides flavonols (quercetin, rutin, kaempferol), flavan-3-ols, and phenolic acids (prominently chlorogenic acid), and distinctively among berries, iridoids (loganic acid, loganin, sweroside), which are rare in *Vaccinium* fruits [62,64,71]. Haskap also tends to show higher vitamin C levels than blueberries across many cultivar/site comparisons [50,64].

### 4.7. Mechanistic Complementarity Between Blueberries and Haskap Berries

The phytochemical profiles described above suggest that blueberries and haskap berries may exert complementary, rather than redundant, biological effects on vascular health. Blueberries (*Vaccinium* spp.) provide a broad spectrum of anthocyanins in which malvidin- and delphinidin-based glycosides are prominent, together with abundant flavonols, phenolic acids and stilbenes such as resveratrol and pterostilbene [13,14,15,16,21,50,51,52,53]. Haskap (*Lonicera caerulea* L.) is instead characterized by very high levels of cyanidin-3-O-glucoside (C3G) as a predominant anthocyanin, along with other anthocyanins, flavonols, phenolic acids and a distinctive profile of iridoids and secoiridoid glycosides [64,65,66,67,68,69,70,71,73,74,75,84].

These compositional differences are mirrored by partially distinct emphases in preclinical and clinical findings. Blueberry interventions have been most consistently associated with improvements in endothelial function and arterial stiffness, together with favorable changes in cardiometabolic biomarkers [13,17,21,22,46,47,48,49,55,56,58,59,60,62,63]. In contrast, the haskap literature, although predominantly preclinical, highlights potent antioxidant and anti-inflammatory actions, hepatoprotective and metabolic effects, and emerging signals on blood pressure and vascular outcomes [64,69,78,79,80,81,84,101,102,103,107]. At a conceptual level, blueberries may be relatively stronger modulators of endothelial NO-related and lipoprotein pathways, whereas haskap berries may provide pronounced C3G- and iridoid-driven antioxidant and anti-inflammatory activity.

The main phytochemical classes in blueberries and haskap relevant to vascular health are summarized in Table 1. The principal anthocyanin species and their typical total contents in blueberries and haskap are summarized in Table 2.

**Figure 1:**
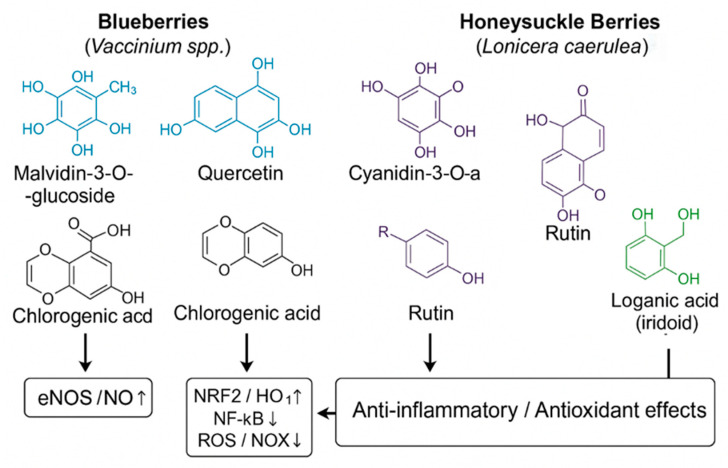
Bioactive compounds and molecular effects of blueberries and honeysuckle berries.

These mechanistic links between phytochemical composition and vascular endpoints are explored in greater detail in Section 5, while the hypothesis of potential additive and synergistic effects arising from combined blueberry–haskap intake is developed in Section 8. The main classes of bioactive compounds and their putative vascular targets are summarized in Figure 1.

## 5. Molecular Mechanisms

### 5.1. NO/eNOS-Mediated Vasodilation

One of the most consistently reported vascular benefits of berry-derived anthocyanins is the enhancement of nitric oxide (NO) signaling via endothelial nitric oxide synthase (eNOS) [57,94]. Blueberry polyphenols, particularly malvidin glycosides, activate PI3K/Akt–eNOS signaling, including phosphorylation at Ser1177 [26], and also display antioxidant actions in endothelial cells [23], which together help limit eNOS uncoupling under oxidative stress [46]. In human aortic endothelial cells, blueberry polyphenol extracts restored angiotensin II-induced reductions in NO and increased NOS activity, resulting in improved vasodilation [26,46]. Animal and clinical studies indicate that chronic blueberry consumption enhances flow-mediated dilation (FMD) and, in some trials, lowers diastolic blood pressure—particularly in individuals with metabolic syndrome [24,49,55]. Honeysuckle berries (*Lonicera caerulea*) appear to exert parallel vascular effects via their dominant anthocyanin, cyanidin-3-O-glucoside (C3G), which may increase NO bioavailability indirectly through AMPK–eNOS signaling and the mitigation of oxidative injury [53,62,71]. Additionally, chlorogenic acid and iridoids present in honeysuckle may help preserve and provide complementary endothelial support [64,71].

### 5.2. NRF2-Dependent Antioxidant Defense

Oxidative stress is a major driver of endothelial dysfunction and atherosclerosis. Anthocyanins and flavonols from blueberries strongly activate NRF2 and induce antioxidant enzymes such as heme oxygenase-1 (HO-1), superoxide dismutase (SOD), catalase, and glutathione peroxidase, while concurrently increasing NO and reducing ROS in endothelial cells [46,56]. In endothelial models, blueberry extracts decrease ROS accumulation, lower the expression/activity of pro-oxidant sources (e.g., xanthine oxidase and NOX4 under hyperglycemic conditions), and support mitochondrial redox homeostasis, thereby protecting NO bioavailability [51,56,57]. In haskap/honeysuckle (*Lonicera caerulea*), the main anthocyanin—cyanidin-3-O-glucoside (C3G)—and its phenolic metabolites (e.g., dihydroxybenzoic acids derived from anthocyanin degradation) are involved in activating antioxidant defenses, with increased HO-1/SOD and improvement of oxidative stress markers in hepatic and cardiovascular models [53,72,101]. The co-presence of iridoids (e.g., loganic acid, loganin)—rare in other fruits—provides an antioxidant “cushion” that complements polyphenols, strengthening endothelial resilience to oxidative insults [62,71].

### 5.3. NF-κB and Inflammatory Signaling

Chronic vascular inflammation underlies the initiation and progression of atherosclerosis [6,81]. In endothelial cells, blueberry anthocyanins—especially malvidin-3-glucoside and malvidin-3-galactoside—suppress NF-κB activation and downregulate adhesion molecules (ICAM-1, VCAM-1) and chemokines such as MCP-1, thereby reducing monocyte adhesion [25,46]. In humans, blueberry interventions consistently improve vascular function, while effects on systemic inflammatory biomarkers (CRP, TNF-α, IL-6) are small and inconsistent across trials; some studies and syntheses report no change, whereas others note modest reductions in selected populations [7,12,98]. Honeysuckle/haskap (*Lonicera caerulea*) shows convergent anti-inflammatory actions in experimental models, with C3G, chlorogenic acid and iridoids attenuating pro-inflammatory signaling (e.g., NF-κB/MAPK) and lowering cytokine outputs, supporting endothelial homeostasis [53,62,71,101,104,107].

### 5.4. Lipoprotein Oxidation and Platelet Function

Blueberry (poly)phenols—including anthocyanins, flavonols, and proanthocyanidins—attenuate low-density lipoprotein (LDL) oxidation by scavenging reactive species and chelating transition metals; in human and *in vitro* models, they also promote macrophage cholesterol efflux, thereby limiting foam-cell formation [20,98,108]. In addition, they blunt platelet aggregation by interfering with thromboxane A–dependent Ca^2^ signaling and with the GPVI/Syk/PLC2 pathway [27,109]. Haskap/honeysuckle (*Lonicera caerulea*) provides complementary protection: its major anthocyanin, cyanidin-3-O-glucoside (C3G), is metabolized in humans to protocatechuic acid and related phenolics (e.g., ferulic/vanillic), which reduce lipid peroxidation and downregulate endothelial adhesion molecules [28,89,99,117]. The berry’s high vitamin C content can further stabilize LDL particles and act synergistically with anthocyanins to protect vascular lipoproteins from oxidative modification [62,64,110].

### 5.5. Metabolic and Gut–Microbiota-Mediated Mechanisms

Despite the high native concentrations of anthocyanins in blueberries and honey-suckle, the systemic bioavailability of intact anthocyanins is very low (typically <1% of intake). Instead, circulating profiles are dominated by smaller microbial and host-derived phenolic metabolites (e.g., protocatechuic, ferulic, vanillic, syringic acids) [89,115]. These metabolites are generally more stable and longer-lived than their parent anthocyanins and show potent vascular bioactivities—including inhibition of NF-κB–driven inflammation and activation of cytoprotective pathways (e.g., eNOS/NO signaling and NRF2) [93,95]. In clinical settings, improvements in endothelial function after blueberry intake correlate most strongly with circulating phenolic metabolites rather than with intact anthocyanins [49,60]. Inter-individual differences in gut microbiome composition shape metabolite production and thus physiological responses—an emerging determinant of berry efficacy—and honeysuckle itself can modulate gut communities *in vivo* [92,95,102,118]. The potential for additive or synergistic vascular effects arising from combined blueberry–haskap intake is discussed in detail in Section 8. Key vascular pathways modulated by blueberries and haskap, together with the level of preclinical and clinical evidence, are summarized in Table 3.

## 6. Preclinical Evidence

Preclinical studies have been instrumental in mapping the molecular pathways through which berry (poly)phenols may influence vascular health. *In vitro* experiments in endothelial and vascular cells show that blueberry anthocyanins and related polyphenols increase eNOS expression and phosphorylation, enhance NO bioavailability, activate NRF2-dependent antioxidant responses, and inhibit NF-κB–mediated expression of adhesion molecules and pro-inflammatory cytokines [23,24,25,26,113,119,120,121,122]. Similar models indicate that haskap-derived C3G and associated phenolics reduce oxidative stress, attenuate inflammatory signaling, and protect endothelial cells and vascular tissues in the context of metabolic and hypertensive stress [69,79,80,81,84,101,102,103,107]. *In vivo*, anthocyanin-rich blueberry and haskap extracts have improved vascular structure and function in hypertensive or obese rodent models, reduced oxidative damage, and favorably modulated lipid and glucose metabolism [28,56,58,62,69,80,84,101,102,103,107,108,123].

However, the physiological relevance of many *in vitro* experiments requires careful consideration. A substantial proportion of cell-based studies employ micromolar concentrations of intact anthocyanin glycosides (often 10–100 μM), which exceed the low nanomolar to low micromolar plasma levels typically observed after realistic dietary intakes in humans [21,27,36,37,38,89,90]. Moreover, anthocyanins are extensively metabolized in the gut and liver, and parent glycosides are only transiently detectable in circulation. Human tracer and kinetic studies show that phenolic acids such as protocatechuic and vanillic acid, as well as conjugated derivatives of C3G, represent major circulating and urinary metabolites [21,27,38,89,90,91]. Several of these metabolites exert vascular effects at concentrations that are closer to those achieved *in vivo*, including improvements in endothelial NO signaling, reductions in oxidative stress, and modulation of adhesion molecule expression and monocyte–endothelial interactions [28,29,30,31,32,110,111,112,123].

Accordingly, the mechanistic framework derived from preclinical work should be interpreted as a hypothesis-generating map of potential targets rather than a direct reflection of *in vivo* pharmacology of intact anthocyanins. Future mechanistic studies should prioritize physiologically relevant exposure scenarios, including realistic concentration ranges, metabolite mixtures that reflect human plasma and urine profiles, and co-culture or organoid models that better capture the vascular–immune–metabolic interface. Nevertheless, the consistency with which blueberry and haskap (poly)phenols modulate NO signaling, oxidative and inflammatory pathways, and lipid- and platelet-related mechanisms across diverse experimental systems provides a coherent scaffold to interpret human intervention data and to formulate testable clinical hypotheses.

## 7. Clinical Evidence

Acute crossover interventions with blueberries demonstrate biphasic, time-dependent improvements in endothelial function, with FMD increasing at approximately one to two hours and again near six hours post-ingestion; importantly, these dynamics track circulating phenolic metabolites, directly linking postprandial biotransformation to vascular reactivity [60]. The observed improvements in flow-mediated dilation are consistent with mechanistic data showing that enhanced eNOS activation and reduced oxidative stress improve endothelial-dependent vasodilation [82]. Over eight weeks in postmenopausal women with elevated blood pressure, daily freeze-dried blueberry powder reduced systolic and diastolic blood pressure and improved indices of peripheral arterial stiffness (for example, brachial–ankle pulse wave velocity), alongside increases in NO bioactivity, a pattern that suggests peripheral arterial beds may respond faster to polyphenol signaling than central elastic arteries over this timeframe [120]. Over six months in adults with metabolic syndrome, a double-blind randomized trial showed improved endothelial function and favorable shifts in cardiometabolic biomarkers with daily blueberry intake, supporting durability of effects beyond the acute window [24,98]. A contemporary quantitative synthesis integrating randomized trials reported a mean FMD increase of about 1.5 percentage points and a reduction in diastolic blood pressure of roughly 2 mmHg with blueberry interventions, with stronger blood-pressure effects observed among smokers; although modest per individual, such effects are meaningful at the population level and are consistent with the mechanistic signature seen in preclinical models [17,24]. Human trials with honeysuckle (haskap) are comparatively early, yet acute testing in older adults has suggested small decreases in diastolic blood pressure and heart rate alongside cognitive benefits after high-C3G dosing, while a randomized trial in recreational runners showed improved 5 km performance with small but significant reductions in submaximal heart rate and oxygen consumption, implying improved hemodynamic efficiency even in the absence of direct endothelial endpoints [63,83]. Taken together with the robust preclinical dossier on C3G and its metabolites, these findings warrant harmonized twelve- to twenty-four-week randomized trials that standardize total anthocyanin delivery, resolve peripheral versus central stiffness with complementary indices (FMD, carotid–femoral and brachial–ankle pulse wave velocity, augmentation index), and integrate metabolic and microbiome profiling to capture inter-individual variability in metabolite signatures and clinical response [17,63,124].

From a practical perspective, anthocyanin doses in blueberry trials typically range from approximately 100 to 600 mg/day, delivered as whole berries, juices or freeze-dried powders, but there is substantial heterogeneity in the food matrices used, the co-occurring bioactives and the characteristics of the study populations. At present, the available evidence supports this range as biologically active and likely safe, yet it remains premature to define a single “optimal” anthocyanin dose for vascular protection. Future mechanism-aware trials should therefore pre-specify anthocyanin targets, systematically explore dose–response relationships and avoid extrapolating quantitative thresholds across studies that differ in design, matrix, and background diet.

### Safety, Tolerability and Potential Interactions

Across randomized blueberry and mixed-berry interventions, daily intakes providing approximately 100–600 mg/day of anthocyanins delivered, as whole berries, juices or freeze-dried powders have generally been well tolerated, with no consistent signal for serious adverse events compared with control conditions [12,17,20,21,24,55,56,58,59,60,62,63]. The most frequently reported side effects are mild and transient gastrointestinal symptoms (e.g., bloating, changes in stool frequency or consistency), typically occurring in the context of high-fiber or juice-based interventions and resolving without sequelae. In populations with metabolic syndrome, pre-hypertension or type 2 diabetes, long-term blueberry intake has not been associated with clinically relevant deterioration of glycemic control, liver function or renal parameters, although most studies have been of limited duration (up to six months) and sample size [21,22,24,58,62,63,82].

Specific safety data for haskap are more limited, but acute and short-term studies in older adults and athletes have not reported serious adverse events at doses designed to deliver high cyanidin-3-O-glucoside (C3G) exposure [69,80,82,83,84,85]. Preclinical work likewise suggests a wide therapeutic window, although formal toxicological assessments and long-term human trials remain sparse [64,69,79,80,81,84,101,102,103,107]. From a mechanistic perspective, the antiplatelet and endothelial-protective actions of anthocyanins and related polyphenols raise a theoretical concern for interactions with antiplatelet or anticoagulant medications, particularly when berry preparations are consumed at high doses in concentrated supplement forms [27,97,98,109,117]. While existing clinical trials have not consistently shown an excess of bleeding events, none have been powered to detect rare safety signals, and most have excluded individuals on intensive antithrombotic regimens. Consequently, until more systematic safety data are available, high-dose berry extracts should be used cautiously in patients receiving antiplatelet or anticoagulant therapy, and future trials should incorporate standardized adverse-event monitoring and predefined safety endpoints.

## 8. Synergy Between Blueberries and Honeysuckle Berries

### 8.1. Mechanistic Complementarity in Phytochemical Profiles

As detailed in Section 3 and Section 4, blueberries and haskap berries share a high overall anthocyanin density but differ markedly in their dominant anthocyanin species and in their non-anthocyanin bioactives. Blueberries typically provide a broad spectrum of anthocyanins in which malvidin- and delphinidin-based glycosides are prominent, alongside abundant flavonols, phenolic acids and stilbenes such as resveratrol and pterostilbene [13,14,15,16,21,33,50,51,52,53]. Haskap berries, in contrast, are characterized by very high levels of C3G as a predominant anthocyanin, together with other anthocyanins, flavonols, phenolic acids and a distinctive profile of iridoids and related secoiridoid glycosides [64,65,66,69,70,71,72,73,74,75,76,84].

This compositional landscape suggests that blueberry and haskap extracts are not redundant but rather engage overlapping yet distinct biological targets. Blueberry-derived anthocyanins and stilbenes have been most consistently linked to improvements in endothelial function, arterial stiffness, insulin sensitivity and lipid handling in human trials [17,21,22,55,56,58,59,60,62,63], whereas haskap-derived C3G, iridoids and other phenolics have shown potent antioxidant, anti-inflammatory and metabolic effects in preclinical models, with emerging signals on vascular and cardiometabolic endpoints [69,79,80,81,84,101,102,103,107]. At a conceptual level, blueberries may be viewed as relatively stronger modulators of endothelial NO-related and lipoprotein pathways, while haskap berries may contribute pronounced anti-inflammatory and metabolic actions via their high C3G and iridoid content.

### 8.2. A Hypothesis for Additive and Synergistic Vascular Effects

On the basis of these complementary phytochemical and mechanistic profiles, we propose a hypothesis of additive—or potentially synergistic—effects on vascular endothelial function when blueberries and haskap berries are consumed together. This hypothesis is currently mechanistic and exploratory rather than empirically proven: no randomized controlled trials have directly compared combined blueberry–haskap interventions with single-berry intakes, and the existing clinical evidence base is substantially stronger for blueberries than for haskap.

In principle, a combination of blueberries and haskap could broaden the coverage of vascular pathways affected by berry (poly)phenols. Blueberry anthocyanins and pterostilbene may preferentially support NO bioavailability, eNOS activation, HDL functionality and protection against LDL oxidation and platelet activation [13,21,23,24,26,27,28,31,32,53,58,59,96,97,98], while haskap C3G and iridoids may exert potent NRF2-mediated antioxidant and NF-κB–modulating effects, attenuate hepatic steatosis and dyslipidemia, and influence gut microbiota composition [69,70,71,79,80,81,84,101,102,103,107]. In individuals with concomitant endothelial dysfunction, low-grade inflammation and metabolic disturbance, such a broadened mechanistic footprint could, in theory, translate into greater improvements in FMD, arterial stiffness and circulating markers of endothelial activation than those observed with either berry alone.

Importantly, the magnitude and direction of any interaction between blueberries and haskap will depend on dose, intake duration, the relative proportion of each berry, the food matrix, gut microbiota composition and background pharmacotherapy. Synergy in this context should therefore be viewed as a working model—supported by the broader literature on multi-component polyphenol interventions and quantitative frameworks for combination effects [86,87,88]—rather than as a conclusion based on direct clinical evidence.

### 8.3. Roadmap for Mechanism-Aware Clinical Trials

To rigorously evaluate the proposed synergy hypothesis, future clinical trials should be explicitly designed to compare blueberry-only, haskap-only and combined interventions. Target populations could include older adults or individuals with metabolic syndrome, pre-hypertension or early stage hypertension, in whom endothelial dysfunction and arterial stiffening are already detectable but potentially reversible. Interventions should use well-characterized, standardized berry matrices (e.g., freeze-dried powders or juices) with clearly defined doses of total anthocyanins, C3G and key co-actives such as pterostilbene and iridoids, reflecting realistic dietary intakes rather than pharmacological exposures [17,21,22,55,56,58,59,62,63,69,80].

Primary vascular endpoints should encompass both functional and structural measures, including brachial artery FMD, carotid–femoral pulse wave velocity (cfPWV) and augmentation index (AIx), complemented by office and ambulatory blood pressure. Secondary and exploratory outcomes could include biomarkers of endothelial activation (e.g., ICAM-1, VCAM-1, E-selectin), oxidative stress, low-grade inflammation (e.g., hsCRP, IL-6, TNF-α), lipid profile, HDL function and platelet reactivity, as well as indices of glucose metabolism and hepatic steatosis where relevant [17,22,28,31,32,56,58,59,69,91,96,97,98,108].

A key feature of mechanism-aware trials will be the parallel characterization of circulating and urinary (poly)phenol metabolites and gut microbiota composition. Such data would allow for the identification of (poly)phenol-related metabotypes and their association with vascular responses to single- versus mixed-berry interventions [21,27,29,30,36,37,38,89,90,91,92,93]. In selected settings, *ex vivo* or *in vitro* assays could be used to quantify additive or synergistic effects of blueberry- and haskap-derived extracts or metabolite mixtures on endothelial cells and platelets using established models such as isobolograms or the Chou–Talalay combination index [86,87,88]. Finally, all combination trials should systematically monitor safety, tolerability and potential interactions with concomitant medications, as long-term high-intake patterns of mixed berry preparations have not yet been extensively characterized in clinical populations. In the longer term, such integrated phenotyping of (poly)phenol metabolites and gut microbiota could support more individualized nutritional strategies, in which specific berry combinations or doses are tailored to metabotype-defined responder groups. At present, however, the evidence base is insufficient to recommend routine metabotype-guided personalization in clinical practice, and these approaches should be regarded as research priorities rather than immediately actionable tools.

## 9. Conclusions and Future Directions

Collectively, current evidence supports incorporating both blueberries (*Vaccinium* spp.) and haskap/blue honeysuckle (*Lonicera caerulea* L.) into cardioprotective dietary patterns, with stronger direct clinical support available for blueberries and a predominantly preclinical yet coherent dossier for haskap [17,34,62,87]. Blueberry interventions consistently improve endothelial function (flow-mediated dilation) and lower diastolic blood pressure in meta-analysis, providing a plausible vascular mechanism for anthocyanin-rich foods [17,24]. Haskap offers a complementary phytochemical matrix—dominated by cyanidin-3-O-glucoside and enriched with iridoids and vitamin C—that aligns mechanistically with antioxidant, anti-inflammatory and metabolic support, although its human evidence base is still limited to a small number of acute and short-term studies [62,69,84,87,88].

At the same time, several limitations of the current literature should be acknowledged. Clinical trials differ substantially in anthocyanin dose, food matrix, duration, and background diet, which complicates quantitative comparisons and dose–response inferences [17,21,55,56,58,59,62,63]. Many mechanistic studies employ supra-physiological concentrations of native anthocyanins rather than physiologically relevant mixtures of circulating metabolites, and long-term safety data for high-dose, standardized berry extracts—especially haskap—remain sparse [21,27,29,30,36,37,38,89,90,92,93]. Furthermore, inter-individual variability in (poly)phenol metabolism and gut microbiota composition is rarely accounted for, despite emerging evidence that “(poly)phenol-related gut metabotypes” may shape vascular responses [21,27,36,92,93]. These gaps underline that the current findings should be interpreted as hypothesis-generating rather than practice-changing and do not yet justify specific, dose-precise recommendations for combined blueberry–haskap supplementation in clinical care.

From a translational perspective, a combined nutraceutical or functional-food strategy is biologically plausible: malvidin-dominant blueberry profiles may complement C3G-rich, iridoid-bearing haskap by broadening the spectrum of circulating phenolic metabolites and molecular targets [33,56,62,69,84,87,88]. To test this synergy hypothesis, future mechanism-aware randomized trials should directly compare blueberry-only, haskap-only and combined interventions, harmonize dosing by total anthocyanins and key index molecules (e.g., C3G), and apply rigorous product characterization and reporting standards for herbal and natural-product interventions [84,85,87,125]. Analytical pipelines should integrate validated HPLC/UPLC-MS profiling and targeted metabolomics to relate exposure and metabolite formation to vascular endpoints, alongside systematic assessment of gut microbiota and “metabotypes” [21,27,36,37,38,85,89,90,92,125]. Finally, process and formulation research should prioritize stability-preserving technologies (freeze-drying, co-pigmentation, micro-/nanoencapsulation, polymer-based carriers) that enhance anthocyanin resilience and bioavailability without compromising safety [34,35,41,42,43,45,54]. Together, such standardized, mechanism-aware trials—integrated with advanced analytics—will be essential to determine whether combining blueberries and haskap yields additive or synergistic vascular benefits that are clinically meaningful at the population level [17,34,45,54,84,85,87,89,125].

## Figures and Tables

**Table 1:** Major phytochemical classes in blueberries (*Vaccinium* spp.) and haskap (*Lonicera caerulea* L.) relevant to vascular endothelial health.

Berry/Source	Phytochemical Class	Representative Compounds	Main Vascular-Relevant Actions	Refs
Blueberries	Anthocyanins	Malvidin-, delphinidin-, cyanidin-, petunidin-, peonidin-glycosides	NO/eNOS activation; antioxidant & anti-inflammatory; improved endothelial function; modest BP reduction	[13,14,16,20,21,23,24,25,26,33,50,52,53,56,94]
Blueberries	Flavonols	Quercetin, myricetin, kaempferol glycosides	Antioxidant; improves NO bioavailability; supports vasodilation	[11,13,14,16,43,46,50,55]
Blueberries	Flavan-3-ols & PACs	Catechin, epicatechin, proanthocyanidin oligomers	Inhibits LDL oxidation; modulates platelets; supports cholesterol efflux	[14,16,20,23,27,50,98,108,109]
Blueberries	Phenolic acids	Chlorogenic, caffeic, ferulic, PCA	Reduces oxidative stress; improves endothelial function; anti-inflammatory	[14,28,89,90,94,110,111,112,113,114]
Blueberries	Stilbenes	Pterostilbene, resveratrol	Antioxidant; anti-inflammatory; eNOS activation; lipid effects	[13,14,16,21,50,52,53]
Blueberries	Vitamins & micronutrients	Vitamin C, vitamin E, trace elements	Antioxidant protection; prevents LDL oxidation; supports redox homeostasis	[11,16,50,115,116]
Blueberries	Dietary fiber & matrix	Fiber, organic acids, sugars	Modulates glycemia/lipemia; gut microbiota interactions	[14,16,21,34,35,36,37,38,48,94]
Haskap	Anthocyanins	C3G, cyanidin-3-rutinoside, cyanidin-3,5-diglucoside	Strong antioxidant/anti-inflammatory; AMPK/eNOS & PPAR-α activation	[64,65,66,67,68,70,71,72,75,76,78,84,85,101,102,103,106,107]
Haskap	Flavonols	Quercetin, rutin, kaempferol	Antioxidant & anti-inflammatory; endothelial support	[64,65,68,79,104]
Haskap	Flavan-3-ols & PACs	Catechin, epicatechin, proanthocyanidins	Antioxidant; oxidative stress & inflammation modulation	[64,75,79,80,81,84]
Haskap	Phenolic acids	Chlorogenic, caffeic-type acids, PCA, vanillic	Antioxidant; AMPK/Nrf2/NF-κB modulation	[64,65,71,72,74,76,101,102,103,106,107]
Haskap	Iridoids	Loganic acid, loganin, sweroside	Anti-inflammatory & antioxidant; metabolic/vascular support	[62,64,65,66,68,71,72,74,75,76,84,85]
Haskap	Vitamins & micronutrients	Vitamin C, vitamin E, minerals	High antioxidant capacity; endothelial protection	[50,64,65,66,67,72]
Haskap	Dietary fiber & matrix	Fiber, organic acids, sugars	Modulates microbiota; impacts metabolism & lipid handling	[64,66,67,74,75,76,84,85,101,102,103,106,107]

**Table 2:** Main anthocyanins in blueberries (*Vaccinium* spp.) and honeysuckle berries/haskap (*Lonicera caerulea* L.): typical composition and content ranges.

Berry/Source	Major Anthocyanins (Predominant Glycosides)	Approximate Total Anthocyanin Content (mg/100 g Fresh Weight) *	Notable Features of Anthocyanin Profile and Differences	Selected References **
Blueberries (*Vaccinium* spp.)	Malvidin-3-glucoside and malvidin-3-galactoside (often predominant); delphinidin-3-glucoside/-galactoside, petunidin-3-glucoside, cyanidin-3-glucoside, peonidin glycosides.	Typically ~80–300 mg/100 g FW across cultivated highbush and lowbush blueberries; wild or strongly pigmented cultivars can reach or exceed ~400–450 mg/100 g FW, depending on genotype, growing conditions and analytical method.	Broad and relatively balanced spectrum of malvidin-, delphinidin- and petunidin-based anthocyanins; pigments mainly localized in the skin. Compared with haskap, blueberry anthocyanins show a higher contribution of malvidin-based glycosides and a somewhat lower proportion of cyanidin-3-O-glucoside (C3G).	[13,14,16,20,21,23,24,25,26,29,30,33,50,52,53,56,94] (or subset used in Section 3.1, Section 3.2, Section 3.3, Section 3.4)
Honeysuckle berries/haskap (*Lonicera caerulea* L.)	Cyanidin-3-O-glucoside (C3G; predominant); cyanidin-3-rutinoside, cyanidin-3,5-diglucoside; pelargonidin-3-glucoside, peonidin glycosides.	Across cultivars, total anthocyanin content commonly ranges from ≈150 up to >650 mg/100 g FW; values around 250–400 mg/100 g FW are frequently reported, with high-C3G genotypes (e.g., ‘Wuhezhen’) showing C3G levels ≈400–500 mg/100 g FW.	Anthocyanin profile is strongly dominated by C3G, leading to very intense dark-purple coloration. Compared with blueberries, haskap tends to have higher total anthocyanin concentrations and a more ‘cyanidin-centric’ profile, often accompanied by substantial contributions from iridoids and phenolic acids in the overall phytochemical matrix.	[39,40,54,64,65,66,67,68,69,70,71,72,75,76,78,84,101,102,103,106,107] (or subset used in Section 4.2, Section 4.3, Section 4.4)

* FW: fresh weight. Ranges are approximate and depend on cultivar, growing conditions, ripeness and analytical method; they are intended to illustrate the relative magnitude of anthocyanin content in the two berries rather than to provide exhaustive compositional data for specific cultivars. ** Reference numbers can be adapted to match the final numbering in the main text; in practice, it is sufficient to reuse the compositional/anthocyanin references already cited in Section 3 and Section 4 and in Table 1.

**Table 3:** Key vascular mechanisms modulated by blueberries (*Vaccinium* spp.) and haskap (*Lonicera caerulea* L.): molecular targets and level of evidence (Increse ↑/Decrease ↓).

Pathway/Endpoint	Key Molecular Targets & Effects	Evidence for Blueberries	Evidence for Haskap
NO/eNOS-mediated vasodilation	↑ PI3K/Akt–eNOS; ↑ eNOS Ser1177; ↑ NO; ↓ eNOS uncoupling	↑ NO & vasodilation in models; RCTs show ↑ FMD & modest DBP reductions	C3G/haskap activates AMPK–eNOS; ↑ NO; BP-lowering in animals; emerging acute BP effects
NRF2-dependent antioxidant defense	↑ NRF2; ↑ HO-1, SOD, catalase, GPx; ↓ NOX4, XO; ↓ ROS	Blueberries activate NRF2; ↓ ROS; improved oxidative-stress markers	C3G/PCA induce HO-1/SOD; antioxidant protection in hepatic & CV models
Inflammatory signaling	↓ NF-κB; ↓ ICAM-1, VCAM-1, MCP-1; ↓ TNF-α, IL-6; MAPK modulation	Malvidin anthocyanins ↓ NF-κB & adhesion molecules; vascular improvements in trials	Haskap/C3G/iridoids suppress NF-κB/MAPK; ↓ cytokines; early vascular inflammation signals
LDL oxidation & atherogenesis	↓ LDL oxidation; ↓ oxLDL; ↑ antioxidant protection; anti-foam cell effects	↓ LDL oxidation; some RCTs show ↓ oxLDL & improved redox status	Haskap/C3G reduce oxidative stress & lipid accumulation; anti-atherogenic potential
HDL function & cholesterol efflux	↑ HDL PON1; ↑ cholesterol efflux	↑ HDL efflux capacity & PON1 in dyslipidemia	Limited direct data; metabolites improve lipid handling in models
Platelet function & thrombosis	↓ platelet activation/aggregation; ↓ TxA2; GPVI/Syk modulation	Blueberries reduce aggregation & TxA2 in human/*ex vivo* studies	No trials; C3G/flavonoids show antiplatelet effects in models
Endothelial barrier & leukocyte adhesion	↓ adhesion molecules; ↓ monocyte adhesion; barrier protection	↓ ICAM-1/VCAM-1 & monocyte adhesion; improved FMD & stiffness	Haskap/C3G reduce adhesion markers; consistent early human vascular signals
Metabolic & microbiota-mediated effects	AMPK activation; PPAR-α; improved insulin sensitivity; microbiota modulation	Improved insulin sensitivity & metabolic markers; metabotype-dependent	Haskap activates AMPK/PPAR-α; ↓ steatosis; modulates microbiota; improves cardiometabolic markers

## Data Availability

Data are contained within the article.
